# Consensus on MR Imaging of Endolymphatic Hydrops in Patients With Suspected Hydropic Ear Disease (Meniere)

**DOI:** 10.3389/fsurg.2022.874971

**Published:** 2022-04-28

**Authors:** Yupeng Liu, Ilmari Pyykkö, Shinji Naganawa, Pedro Marques, Robert Gürkov, Jun Yang, Maoli Duan

**Affiliations:** ^1^Department of Otorhinolaryngology-Head and Neck Surgery, Xinhua Hospital, Shanghai Jiaotong University School of Medicine, Shanghai, China; ^2^Ear Institute, Shanghai Jiaotong University School of Medicine, Shanghai, China; ^3^Shanghai Key Laboratory of Translational Medicine on Ear and Nose Diseases, Shanghai, China; ^4^Hearing and Balance Research Unit, Field of Otolaryngology, Faculty of Medicine and Health Technology, School of Medicine, Tampere University, Tampere, Finland; ^5^Department of Radiology, Nagoya University Graduate School of Medicine, Nagoya, Japan; ^6^Unit of Otorhinolaryngology, Department of Surgery and Physiology, University of Porto Medical School, Porto, Portugal; ^7^ENT Centre at Red Cross Square, University of Munich, Munich, Germany; ^8^Ear Nose and Throat Patient Area, Trauma and Reparative Medicine Theme, Karolinska University Hospital, Stockholm, Sweden; ^9^Division of Ear, Nose and Throat Diseases, Department of Clinical Science, Intervention and Technology, Karolinska Institutet, Stockholm, Sweden

**Keywords:** Meniere's disease, magnetic resonance imaging, endolymphatic hydrops, consensus, gadolinium

## Abstract

Endolymphatic hydrops (EH) is considered the histological hallmark of Meniere's disease. Visualization of EH has been achieved by special sequences of inner ear magnetic resonance imaging (MRI) with a gadolinium-based contrast agent *via* intravenous or intratympanic administration. Although it has been applied for more than 10 years since 2007, a unified view on this technique has not yet been achieved. This paper presents an expert consensus on MRI of endolymphatic hydrops in the following aspects: indications and contra-indications for patient selection, methods of contrast-agent administration (intravenous or intratympanic), MRI sequence selection, the specific scanning parameter settings, and standard image evaluation methods and their advantages and disadvantages. For each part of this consensus, a comment is attached to elucidate the reasons for the recommendation.

## Introduction

Meniere's disease (MD) is a disease complex of multifactorial etiology. As no objective methods exist for diagnosis, the Committee on Hearing and Equilibrium of the American Academy of Otolaryngology-Head & Neck Surgery (AAO-HNS) suggests the use of symptom-based guidelines for the diagnosis of MD (1) which has been supported by the Barany Society (2). MD is believed to originate in the inner ear, and endolymphatic hydrops (EH) can be demonstrated in histological preparations or with MRI, though the etiology of the disease is unknown (3) and most likely multifactorial. EH is considered the histological hallmark of MD. In the early days, histopathological post-mortem studies were considered the only way to confirm the diagnosis of MD (4). In the past decades, direct visualization of EH in living subjects was achieved by special sequences of inner ear magnetic resonance imaging (MRI) with a gadolinium-based contrast agent (GBCA) *via* intravenous, intratympanic, or their combined administration with 3 Tesla magnetic resonance imaging (3T MRI) (5). Clinicians have used different MRI algorithms and visualization methods to confirm and classify EH in MD patients (6). In the clinical guideline proposed by the Japan Society for Equilibrium Research in 2020, EH on MRI was regarded as an objective sign for “certain” MD in the diagnostic criteria of MD (7). As a result of the accumulated experience with EH imaging in patients with symptoms of inner ear disorders, the concept of hydropic ear disease (HED) was developed, unifying the various clinical manifestations in patients with EH as well as the primary and secondary etiologies of EH into one comprehensive taxonomy (8–10).

The endolymphatic space forms a closed liquid circulation system, which is separated from the perilymph. After intratympanic injection or intravenous administration, GBCA is absorbed through the round and oval windows, or the blood labyrinth barrier, respectively, and distributed in the perilymph fluid after entering the inner ear. By changing the water proton relaxation rate of local tissues, GBCA can enhance the image contrast ratio between gadolinium-containing tissues and the gadolinium-free tissues to reflect the morphological changes of the surrounding structures (11). Since GBCA primarily enters the perilymphatic space and not the endolymphatic space, the image of the perilymph fluid can be distinguished from the endolymph fluid by looking at the presence (perilymphatic space) and absence (endolymphatic space) of GBCA in the inner ear (12). Currently, 3-dimensional fluid-attenuated inversion recovery (3D-FLAIR) and 3-dimensional real inversion recovery (3D-real IR) sequences are most widely used with various scan parameters. Regarding grading of EH, different grading scales for evaluating the degree of EH have been proposed. Prior to the upcoming 8th International Symposium on Meniere's Disease and Inner Ear Disorders, shifted to occur in April 2023, an international consensus group of experts in inner ear imaging has come together to work out recommendations for the use of endolymphatic hydrops imaging. This consensus includes the following aspects: contrast agent selection, application of contrast agent, indications and contraindications, MR sequence(s), scan parameters, and image evaluation.

## Consensus of the Committee

### Indications/Contra-Indications

Patients who fulfill the 1995 AAO-HNS criteria of “possible,” “probable,” or “definite” MD or patients who fulfill the Barany Society criteria of “probable” or “definite” MD can be included. Patients with fluctuating symptoms of inner ear dysfunction without a definite diagnosis despite specialized neurotological function testing and conventional cranial MRI may be candidates for EH imaging, depending on the therapeutic consequences of a potentially confirmed diagnosis of HED. Both patients suffering from an acute attack of vertigo and patients in a stable stage are eligible for MRI examination (13). Patients enrolled in clinical trials concerned with the therapeutic efficacy of interventions in HED should receive EH imaging as one of the trial outcome parameters. Furthermore, EH imaging is recommended before invasive and/or destructive treatments such as intratympanic gentamicin injections, endolymphatic sac surgery, semicircular canal occlusion, labyrinthectomy, and vestibular neurectomy (14, 15). However, patients who meet MRI contraindications due to mental or drug incompliances should not undergo MRI examination. The incidence of adverse reactions to GBCA is low, occurring in approximately one in 10,000–40,000 injections. Severe, life-threatening anaphylactoid reactions to GBCA are rare. Intravenous application of GBCA should be forbidden in patients with severe gadolinium allergy, severe chronic kidney disease, and acute renal injury (16). Intratympanic application of GBCA should be used cautiously in these patients as well but those with kidney problems could tolerate the very low amount of intratympanic GBCA. We recommend patients with a history of chronic otitis media, otitis media with effusion or tympanic membrane perforation, or with a history of middle ear surgery for intravenous application of GBCA before MRI examination. Patients who are severely overweight or suffer from claustrophobia may hinder imaging with 3T MRI scanners. The clinician should weigh the importance of an MRI-confirmed diagnosis of EH against the potential risks in each individual patient.

#### Comments

The symptoms of MD are diverse. Some patients have symptoms of vertigo and/or types of hearing loss different from those in the currently recommended clinical MD criteria (AAO-HNS 1995 and Barany Society), and they do not strictly meet the current diagnostic criteria. The reason for variability in complaints is not well-understood though it is reasonable to assume that the variability may be associated with different genotypes, comorbidities, and primary vs. secondary etiologies (8). For these patients, inner ear imaging technology can help clinicians to identify whether it is a disease related to EH, to provide a reference for an informed choice of treatment options.

### Type and Application of Contrast Agents

Commonly used MRI contrast agents include gadoterate megluminate, gadobutrol, gadobenate dimeglumine, gadopentetic acid dimeglumine, and gadodiamide. All of the contrast agents mentioned above, except gadoterate megluminate, were reported to be safe for intratympanic and intravenous injection (17–24). There are still insufficient data about the effect of contrast agent type on the quality of MRI images aimed at detecting EH. From clinical experience, not many differences in image quality seem to be present among contrast agents. We therefore recommend that currently, clinicians do not need to pay special attention to the type of gadolinium contrast agents. However, further research is needed on this topic.

Generally, both intratympanic and intravenous injection of GBCA can be used. Based on the existing clinical experience and clinical safety studies, we recommend that the contrast medium should be diluted eightfold in saline solution before intratympanic injection (25). However, it has not been established yet whether higher concentrations of GBCA intratympanically applied may cause ototoxicity in the clinical application. The tympanic cavity should be filled with the contrast agent for better absorption through the oval window and round window. Before injection of the GBCA, an anterior-superior puncture of the tympanic membrane should be performed, creating an “overpressure valve” for the middle ear gas during the injection of the GBCA and avoiding excessive middle ear pressure build-up which may cause pain and transient vertigo in the patient. For the injection, an ultra-thin cannula with a diameter of 0.4 mm is recommended in order to avoid a potentially persisting perforation of the ear drum (26). Clinicians should ask patients to remain in a supine position with the head turned by 45 degrees toward the contralateral side for 30 min after injection. Speaking and swallowing should be avoided as much as possible during this period. MRI is recommended to be performed 24 h after the intratympanic administration (27, 28).

Intravenous GBCA administration is also a suitable route of delivery. A single intravenous dose of GBCA (0.1 mmol/kg body weight) should be administered intravenously. Intravenous administration of double-dose GBCA might be considered when the pulse sequence optimization is not mature enough to visualize EH with single-dose GBCA. However, taking into account the gadolinium deposition issue in the brain, a single dose of macrocyclic-type agents is recommended (29), especially in patients undergoing multiple EH imaging evaluations such as participants in clinical trials. Furthermore, the use of a double dose of GBCA is not approved in some countries. MRI is recommended to be performed 4 h after intravenous application of GBCA (30, 31).

#### Comments

Currently, there is a shortage of high-quality studies to compare the visualization of the inner ear between intratympanic and intravenous dosing of GBCA, though general opinion suggests better imaging with the intratympanic approach (32, 33). However, the intratympanic method has some restrictions as GBCA is not registered for intratympanic use by national pharmaceutical agencies. An appropriate approach should be chosen with consideration of the clinical characteristics of each patient. Some patients would not accept intratympanic injection when they have access to the intravenous method as an alternative. Due to the difference of the permeability of the round and oval windows, the signal intensity of the perilymph after intratympanic injection may have larger inter-individual differences than that after intravenous injection (34). Also, a single intratympanic injection to the affected side would not enable bilateral observation of both ears. Duan et al. first reported in 2004 that using round window application of GBCA showed no affection in auditory brainstem response thresholds in animal study, indicating that gadolinium is non-toxic to the guinea pig cochlea (24). Intratympanic administration of GBCA has also been reported to be well-tolerated in humans (35–37). The application of intratympanic injection is limited in patients with some diseases such as external ear malformation, acute otitis media, and tympanic membrane perforations.

### MRI Sequence and Scanning Parameters

We recommend the 3D-FLAIR sequence and 3D-real IR sequence (38) as a basic imaging sequence that can characterize the signal differences between the contrast-enhanced perilymph and non-contrast-enhanced endolymph. Subtraction of two kinds of images with slightly different inversion time is frequently employed to produce 3D-real IR images (39). One is 3D-FLAIR, which provides a positive perilymph signal. The other employs a shorter inversion time to produce a positive endolymph image (40). The subtraction of these two kinds of images is called a HYDROPS (HYbriD of Reversed image Of Positive endolymph signal and native image of positive perilymph Signal) image. Many reports with hydrops images can be found using single-dose intravenous GBCA (41, 42). The advantage of the 3D-real IR sequence is that clinicians are able to identify the signals from the endolymph space, perilymph space, and surrounding bone tissues on one single unprocessed 3D-real IR image. The endolymph space and surrounding bone tissues cannot be separated using the 3D-FLAIR sequence. However, the 3D-FLAIR sequence is superior to the 3D-real IR sequence in cases where GBCA was insufficiently distributed into the perilymphatic space after an intratympanic injection (32). 3D-real IR imaging now can be performed even with single-dose intravenous GBCA (43).

Repetition time (TR), echo time (TE), inversion time (TI), readout flip angle (FA), field of view (FOV), slice thickness, and matrix size are the main scan parameters in the MRI of EH. Different parameters were previously proposed by clinicians from different medical centers in the world. For intratympanic GBCA administration, when the GBCA concentration in the labyrinth is high, the adjustments of pulse sequence parameters are not so strict. However, for single-dose intravenous administration, parameters should be strictly defined. Otherwise, meaningful results cannot be expected. Slight changes of the parameters might ruin the entire study. Successful parameters can be found in a previous review paper (32).

#### Comments

Clinicians, radiologists, and MRI technicians could adjust the parameters depending on the actual situation in the MR examination to acquire acceptable EH images, however, a test scan and verification are necessary before the clinical study if the newly adjusted protocol is applied.

### How to Evaluate Images

Several grading systems were proposed to visually evaluate and compare the relative areas of the non-enhanced endolymphatic space vs. the contrast-enhanced perilymph space. The classic three-grade scale proposed by Nakashima is most commonly used in current literature. In this grading system, the vestibule and cochlea are analyzed separately (44). Regarding the cochlea, no hydrops is present when the Reissner's membrane remains *in situ* between the endolymph-containing scala media and perilymph-containing scala vestibuli. A mild hydrops is defined by a slight displacement of Reissner's membrane without exceeding the area of the scala vestibule. A significant endolymphatic hydrops is present when the area of the scala media is larger than that of the scala vestibuli. It is recommended to evaluate the axial plane of the cochlea in MRI so as to maximize the visualization of the three turns of the cochlea. Concerning the vestibule, no hydrops is present when the ratio of the endolymphatic area over the sum of the endolymphatic and perilymphatic areas is <1/3. A mild hydrops is present when the ratio of the endolymphatic area over the whole vestibular fluid space ranges between 1/3 and 1/2. A significant hydrops is present when the ratio of the endolymphatic area exceeds 1/2 (44). This classification method is based on the temporal bone specimen study where the area ratio of endolymphatic space to the vestibular fluid ranged from 26.5 to 39.4% (mean 33.2%). This proportion is also confirmed by endolymphatic space imaging in healthy volunteers (29). Based on this three-stage grading, a modified four-stage grading of cochlear hydrops has then been proposed by Gürkov et al. (45, 46): grade 0 = the endolymph is not/hardly visible, grade 1 = the endolymph is clearly visible as round hypointense regions, grade 2 = the perilymph is further displaced by the endolymph but the perilymph still has a crescent appearance, grade 3 = a perilymph with a flat appearance.

Extension of EH into the semicircular canals (SCC) was first observed by Gürkov et al. (47, 48) and linked to caloric hypofunction to the SCC, but the pathophysiological significance of this EH feature is still not entirely resolved.

Another semi-quantitative grading system called “SURI” (saccule to utricle ratio inversion) is proposed as a marker of EH. In this grading system, grade 0 is defined when no saccular abnormality is observed (SURI <1). Grade 1 is defined when SURI≥1. Grade 2 is defined when the saccule is not visible (49). The three-grade scale of cochlea hydrops and four-grade scale of vestibular hydrops proposed by Bernaerts et al. might be considered as a combination of Nakashima's system and the “SURI” system (18). In the evaluation of cochlea hydrops, each grade is defined based on the location of Reissner's membrane. A normal vestibule is defined when the saccule and utricle are visibly separately and take up less than half of the surface of the vestibule. Vestibular hydrops grade I is defined when the saccule becomes equal or larger than the utricle. Vestibular hydrops grade II is defined when there is a confluence of the saccule and utricle, with still a peripheral rim enhancement of the perilymphatic space. Vestibular hydrops grade III is defined when perilymphatic enhancement is no longer visible. This grading system could provide an accurate description of the severity of EH in different parts of the otolith organs.

Based on this vestibular EH grading, a four-stage grading for EH using two axial images/slices has been proposed for use in EH imaging with a 1.5 Tesla scanner and intratympanic GBCA administration. This grading takes into account the more inferior location of the sacculus with respect to the utriculus and the general predilection for vestibular EH to affect the sacculus in the earliest stage of disease evolution: grade 0 = the sacculus in the inferior vestibulum is not/hardly visible; grade 1 = the sacculus in the inferior vestibulum appears with a round shape; grade 2 = endolymph in the inferior vestibulum has completely displaced the perilymph, but the superior vestibulum still has a clear perilymph signal; grade 3 = the perilymph signal is virtually lost on both slices (50).

Inui et al. (51–53) proposed a quantitatively 3D measurement to evaluate the volume of the inner ear endolymphatic space (ELS) in a more accurate way. Positive perilymph images (PPI) and positive endolymph images (PEI) were transferred, and PEI images were subtracted from the PPI images and the images were reconstructed using a specialized workstation. Accurate measurement of EH is helpful in the further study of the relationship between EH and the clinical manifestation and functional results of MD.

#### Comments

Currently, quantification of the degree of cochlear hydrops is still difficult because the cochlear endolymphatic space is divided into different cochlear turns, and each space is quite small. The existing imaging technology is not enough to fully distinguish the cochlear endolymphatic space of all the cochlear turns, especially the apical turns. The semi-quantitative classification system based on the location of Reissner's membrane, which was first proposed by Nakashima, is still considered to be the most convenient method for the evaluation of cochlear hydrops. This classification allows for the visualization of EH in subjects without MD maybe indicating that the endolymphatic space in living organisms is not as tightly regulated as suggested (as also pressure of the eyes) (6). However, evaluation of the vestibule with a high sensitivity for EH specific for MD can be achieved with this method. The Gürkov classification seems suitable for rapid clinical assessment, being based on typical morphological features of different degrees of EH severity. The “SURI” grading system and Bernaerts system have their advantages in vestibular hydrops evaluation. However, these EH grading methods evaluate EH in the cochlea and vestibule separately without considering the extent of the endolymph space distension throughout the entire inner ear. Also, evaluation of semicircular canal EH is not included. Recently, He et al. (54) established a 2D volume-referencing EH grading system in which the volume ratio and the semicircular canals are taken into consideration to better represent the total EH of inner ears. Clinicians can combine the results of MRI and audio-vestibular function tests (electrocochleogram (ECochG), cervical vestibular evoked myogenic potentials (cVEMP), and ocular vestibular evoked myogenic potentials (oVEMP) to evaluate the severity of the disease comprehensively. Also, as indicated above, focusing on quantitative saccule hydrops and utricle hydrops changes for individual patients in a longitudinal imaging study design would provide valuable information for further understanding of the pathophysiological changes in MD patients. A histopathologic study revealed that hydrops initially involves the cochlear duct and the saccule. With the progression of pathology, the utricle and semicircular canals will be involved subsequently (55).

## Author Contributions

JY and MD contributed to the study design and critically reviewed and approved the final manuscript. YL contributed to the detailed study design, drafting of the manuscript, and revised the manuscript. IP, SN, PM, and RG critically reviewed the manuscript. All authors agreed to be accountable for the content of the work, contributed to the article, and approved the submitted version.

## Conflict of Interest

The authors declare that the research was conducted in the absence of any commercial or financial relationships that could be construed as a potential conflict of interest.

## Publisher's Note

All claims expressed in this article are solely those of the authors and do not necessarily represent those of their affiliated organizations, or those of the publisher, the editors and the reviewers. Any product that may be evaluated in this article, or claim that may be made by its manufacturer, is not guaranteed or endorsed by the publisher.
